# It Is Belief in Dualism, and Not Free Will, That Best Predicts Helping: A Conceptual Replication and Extension of Baumeister et al. (2009)

**DOI:** 10.1177/01461672221137209

**Published:** 2023-01-11

**Authors:** Oliver Genschow

**Affiliations:** 1 Leuphana University Lüneburg, Germany

**Keywords:** free will belief, belief in dualism, belief in determinism, helping, pro-social behavior

## Abstract

Previous research found that experimentally reducing people’s belief in free will affects social behaviors. However, more recent investigations could not replicate several findings in this literature. An explanation for the mixed findings is that free will beliefs are related to social behaviors on a correlational level, but experimental manipulations are not able to detect this relation. To test this interpretation, we conceptually replicated and extended a landmark study in the free will belief literature originally conducted by Baumeister et al. In five studies (total *N* = 1,467), we investigated whether belief in free will predicts helping behavior in comparison to other beliefs related to free will. Overall, our results support the original findings, as belief in free will correlated with helping behavior. However, the results also show that the best predictor of helping behavior is not belief in free will but belief in dualism. Theoretical implications are discussed.

Does it matter if people believe in some kinds of meta-physical properties? Over the past few decades, an increasing number of psychologists have become interested in laypeople’s beliefs about meta-cognitive concepts and how these beliefs relate to social behavior. In this respect, a large body of research investigated the social consequences of laypeople’s beliefs surrounding free will (for a review, see Ewusi-Boisvert & Racine, 2018). A landmark article in this literature is Baumeister and colleagues’ (2009) paper published in the journal *Personality and Social Psychology Bulletin* (646 citations on Google Scholar as of October 12, 2022). In two studies, Baumeister et al. found that the endorsement of free will belief relates to individuals’ willingness to help others. Strikingly, Baumeister et al.’s findings have never been replicated. Since recent research casts doubt on the general link between free will beliefs and social behavior (e.g., Genschow et al., 2022), it is unclear whether Baumeister et al.’s findings would withstand a high-powered conceptual replication. Moreover, as Baumeister et al. did not control for beliefs other than free will, it remains open whether belief in free will or other beliefs surrounding free will better predict helping behavior. To fill this gap, we conducted five high-powered conceptual replications and extensions of Baumeister et al.’s Studies 1 and 2.

## Beliefs Surrounding Free Will

Philosophical definitions regarding the concept of free will reached a high level of sophistication within the last decades (Carey & Paulhus, 2013). Therefore, researchers started assessing laypeople’s concepts of free will and the degree to which they believe in these concepts (e.g., Nichols, 2006). The results of such analyses indicate that belief in free will is a metacognitive judgment about the extent to which individuals can control their behavior (Paulhus & Carey, 2011) and are responsible for their actions (Carey & Paulhus, 2013).

Related to belief in free will is belief in determinism. Belief in determinism is linked to the assumption that given the past and the laws of nature, there is only one possible future at any moment in time (e.g., Van Inwagen, 1983). How exactly the belief in determinism is related to belief in free will is part of an ongoing debate in philosophy and psychology. Incompatibilists assume that free will and determinism are the endpoints of the same continuum. Such a conceptualization suggests that the more strongly someone believes in free will, the less (s)he believes in determinism (Rakos et al., 2008; Viney et al., 1982). Interestingly, when measuring both beliefs in laypeople, they are often not correlated (e.g., Nadelhoffer et al., 2014; Wisniewski et al., 2019), suggesting that if there is a link in lay concepts of free will and determinism, it is rather weak or nonexistent. This means that laypeople believe that certain things in life are determined. At the same time, they think they have free choice over their behaviors. These experiences are in line with a compatibilistic view according to which free will and determinism are independent constructs. Based on this idea, persons believing in determinism assume that people could nevertheless be free. Past research has shown that a compatibilistic view might be more widespread in the general public than has been assumed (e.g., Monroe & Malle, 2010; Murray & Nahmias, 2014; Nadelhoffer et al., 2014; Nahmias et al., 2006; Nichols, 2004, 2006; Nichols & Knobe, 2007; Rose & Nichols, 2013; Shepard & Reuter, 2012; Shepherd, 2012). Clark et al. (2019) explain these findings with the notion that people report that free will is compatible with determinism because they desire to uphold moral responsibility.

Another belief closely linked to free will is the belief in dualism. Belief in dualism is often considered a cornerstone of complex beliefs such as the belief in gods and religious belief as a whole (e.g., Bloom, 2007). It reflects the idea that there are multiple forms of reality and that people have nonphysical souls or minds that cannot be reduced to their brains and survive after their bodies die (Nadelhoffer et al., 2014). Thus, it is not surprising that people who strongly believe in dualism typically believe in the existence of a soul-like construct that survives bodily death (Bering, 2006; Boyer, 2001).

## Relation to Social Behavior

Research on the relation between beliefs related to free will and social behavior has largely neglected investigating the role of belief in dualism. Among the few investigations, Burgmer and Forstmann (2018; see also Forstmann et al., 2012) detected a negative relation between belief in dualism and health-related behavior. Other research found a positive association between belief in dualism and teleological reasoning, belief in god, belief in the paranormal, and self-reported purpose in life (Willard & Norenzayan, 2013).

The majority of research investigating associations between beliefs related to free will and social behaviors focused on belief in free will. A large body of this research defined belief in free will as the endpoint of a continuum between belief in determinism and belief in free will. Such research indicated that people’s belief in free will predicts performance in work contexts (Stillman et al., 2010) and academic settings (Feldman et al., 2016). Other studies found that belief in free will is related to perceiving behavior as intentionally driven (Genschow et al., 2017, 2019; Genschow & Lange, 2022; for a review, see Genschow & Brass, 2022), punishing immoral behavior (Clark et al., 2014; Genschow et al., 2017; Martin et al., 2017), rewarding moral behavior (Genschow et al., 2017), as well as blaming victims for their bad luck (Genschow & Vehlow, 2021).

Studies applying experimental manipulations with the aim to reduce individuals’ beliefs related to free will indicated that merely reading texts claiming that free will is an illusion influences social behavior (Baumeister et al., 2009; Protzko et al., 2016; Vohs & Schooler, 2008; Zhao et al., 2014). In this respect, some of the most influential studies were conducted by Baumeister and colleagues (2009). In Study 1 (*N* = 64), Baumeister et al. (2009) applied a Velten-like technique to manipulate participants’ belief in free will. That is, depending on the condition, participants read 15 sentences that were related to free will, to determinism, or neither of these concepts. Afterward, participants were presented with six scenarios in which a person sought help. For each scenario, participants indicated on 9-point rating scales how likely it was that they would help the person. The results indicated that participants who read the deterministic sentences were less likely to help than participants who read the free will sentences or sentences unrelated to free will and determinism. In Study 2 (*N* = 52), Baumeister et al. applied a correlational approach in which participants indicated their belief in free will and the number of hours (from 1 to 9) they would be willing to help another person in need. In addition, the researchers measured participants’ current mood. The results indicated that belief in free will predicted intended helping behavior even when controlling for mood.

Interestingly, despite their widespread impact on the literature, the Baumeister et al. (2009) studies have never been replicated. However, recent research trying to replicate related findings at the intersection of free will beliefs and social behavior was not able to replicate the general finding that experimental manipulations of free will beliefs influence social behavior (Monroe et al., 2017; Nadelhoffer et al., 2020; Open Science Collaboration, 2015; Schooler et al., 2014; Zwaan, 2014). Interestingly, in an effort to replicate Vohs and Schooler’s (2008) finding that free will belief manipulations influence cheating, Buttrick et al. (2020) found indications that participants’ suspiciousness about the manipulation may counteract the effectiveness of free will belief manipulations. Taken together, the above-reviewed studies indicate that landmark studies in the free will belief literature are difficult to replicate.

A current meta-analysis (Genschow et al., 2022) supports this interpretation. Although the meta-analysis found that common anti-free will manipulations reliably reduce beliefs related to free will, it also demonstrated that these manipulations do not affect social behavior. There are several potential explanations for the latter finding. First, the meta-analysis shows that typical free will belief manipulations produce rather weak effects on the manipulation check (i.e., belief in free will). Indeed, given that any effect of free will belief manipulations on attitudes, behavior, and thoughts is most likely smaller than their effect on the beliefs they aim to change (Webb & Sheeran, 2006), detecting downstream consequences is particularly challenging when the effect on the manipulation is already small. Second, standard free will belief manipulations are rather unspecific in the sense that they do not only influence belief in free will but also other (psychological and cognitive) variables and beliefs (Genschow et al., 2022). This is problematic because the different variables influenced by the manipulation could counteract the influence of free will beliefs on the dependent variable, making it particularly challenging to detect downstream consequences. Third, it may be that free will beliefs are not related to social behavior. To put this latter explanation to a critical test, we conducted five high-powered studies in which we assessed the correlation between beliefs surrounding free will and helping.

## Present Research

Taken together, recent research casts doubt on the influence of experimental free will belief manipulations on social behavior (Genschow et al., 2022; Monroe et al., 2017; Nadelhoffer et al., 2020; Open Science Collaboration, 2015; Schooler et al., 2014; Zwaan, 2014). This does not mean, however, that on an interindividual level beliefs surrounding free will are not related to social behavior. It could well be that the existing experimental manipulations are not able to detect a relation that is present on an interindividual level. Hence, two important questions arise: First, can the link between belief in free will and helping originally detected by Baumeister et al. (2009) be conceptually replicated in a correlational study? And second, given that belief in free will and belief in dualism are related to each other, which of these beliefs predict helping above and beyond the other belief? To answer these questions, we conducted five high-powered conceptual replications and extensions of Baumeister et al.’s Studies 1 and 2. By running conceptual replications of one of the landmark findings in the free will belief literature, we contribute to the current call for more replications in psychological research (e.g., Open Science Collaboration 2015) and the call to conceptually replicate (Stroebe & Strack, 2014) important and influential findings (Genschow et al., 2021).

We report all studies we ever conducted in this line of research, all measures, and exclusions. The materials, data, analysis scripts, and a codebook for interpreting the data files are available on the Open Science Framework (OSF; https://osf.io/twxkz/).

## Study 1

### Method

Study 1 was a conceptual replication of Baumeister et al.’s (2009) Study 1. We preregistered the study at Aspredicted (https://aspredicted.org/7r8wn.pdf).

#### Participants

We recruited participants via email. Based on the recommendations for stable correlations put forward by Schönbrodt and Perugini (2013) we aimed to recruit at least 250 participants. In the end, 355 participants (225 female; 126 male; 1 diverse; 2 preferred to self-describe; 1 preferred not to answer) with an age ranging from 16 to 78 (*M* = 31.35; *SD* = 16.75) agreed to take part in the study.

#### Procedure

Participants took part in the study online. First, participants filled in the German translation (Genschow et al., 2019) of the Free Will Inventory (FWI; Nadelhoffer et al., 2014) on 7-point rating scales (1 = *strongly disagree*; 7 = *strongly agree*). The FWI constitutes 15 items. Five items measure the belief in free will (sample items: “People always have the ability to do otherwise,” “People always have free will”), 5 items measure the belief in determinism (sample items: “Everything that has ever happened had to happen precisely as it did, given what happened before,” “Every event that has ever occurred, including human decisions and actions, was completely determined by prior events”), and 5 items measure the belief in dualism (sample items: “The fact that we have souls that are distinct from our material bodies is what makes humans unique,” “Each person has a non-physical essence that makes that person unique”). A full list of the items can be accessed at the OSF (https://osf.io/twxkz/). Cronbach’s alpha was α = .66 for the free will subscale, α = .71 for the dualism subscale, and α = .70 for the determinism subscale.

Second, in line with Baumeister et al. (2009), we measured participants’ mood with the German version (Krohne et al., 1996) of the Positive and Negative Affectivity Schedule (PANAS; Watson et al., 1988). To prepare data for analyses, we reverse coded the items measuring negative mood and then averaged across all items. High values indicate positive mood. Cronbach’s alpha for the PANAS was α = .83.

Third, to measure helping, we presented participants with 10 scenarios. Each scenario described a situation in which a person sought help. For each situation, participants indicated on a 9-point rating scale (1 = *not at all likely*; 9 = *very likely*) the likelihood that they would help in that situation. From the original Baumeister et al. (2009) paper, we could only reconstruct two scenarios (e.g., a homeless person asking for money; someone asking to use a cellphone) because no further information on the other scenarios were provided. For the remaining eight scenarios, we adapted scenarios validated by Gaesser and Schacter (2014). We provided the exact wording of each scenario on the OSF (https://osf.io/twxkz/). To prepare data for analyses we computed for each participant the average across all scenarios (Cronbach’s α = .68). At the end, participants indicated their age and gender (*female; male; diverse; prefer to self-describe; prefer not to answer*).

### Results

To test whether beliefs related to free will correlate with intended helping behavior, we ran preregistered correlational analyses (for an overview, see Table 1). The results indicated that belief in free will (*r* = .13, *p* = .012, 95% confidence interval [CI] = [.03, .23]) and belief in dualism (*r* = .26, *p* < .001, 95% CI = [.16, .36]) but not belief in determinism (*r* = −.05, *p* = .389) correlated with indicated helping behavior. There was also a positive correlation between helping behavior and mood (*r* = .12, *p* = .022, 95% CI = [.02, .22]).

**Table 1.** Intercorrelations Between Helping Behavior, Beliefs Related to Free Will, Mood, and Religiosity in Studies 1–5.

Study & Variable	*M* (*SD*)	1	2	3	4	5	6	7	8	9	10
Study 1 (*N* = 355)											
Helping (1)	6.22 (1.09)	–									
Belief in free will FWI (2)	4.42 (1.11)	.13*	–								
Belief in determinism FWI (3)	3.37 (1.22)	−.05	.13*	–							
Belief in dualism FWI (4)	4.53 (1.21)	.26***	.29***	.17**	–						
Mood (5)	3.60 (0.45)	.12*	.24***	−.02	.05	–					
Study 2 (*N* = 359)											
Helping (1)	2.76 (2.58)	–									
Belief in free will FWI (2)	4.26 (1.25)	.07	–								
Belief in determinism FWI (3)	3.32 (1.18)	−.03	.16**	–							
Belief in dualism FWI (4)	4.42 (1.30)	.12*	.29***	.17**	–						
Mood (5)	3.52 (0.46)	.07	.08	−.003	.09	–					
Study 3 (*N* = 253)											
Helping (1)	4.49 (2.16)	–									
Belief in free will FWI (2)	5.28 (1.12)	.09	–								
Belief in determinism FWI (3)	4.96 (1.44)	.20**	.52***	–							
Belief in dualism FWI (4)	5.17 (1.28)	.22***	.57***	.60***	–						
Mood (5)	3.54 (0.67)	.004	.05	−.21***	−.06	–					
Study 4 (*N* = 251)											
Helping (1)	4.64 (2.30)	–									
Belief in free will FWI (2)	5.48 (1.00)	.01	–								
Belief in determinism FWI (3)	5.10 (1.45)	−.12	.60***	–							
Belief in dualism FWI (4)	5.41 (1.17)	.04	.66***	.65***	–						
Belief in free will FAD (5)	3.86 (0.67)	−.03	.73***	.44***	.58***	–					
Belief in scientific determinism FAD (6)	3.70 (0.71)	−.05	.54***	.75***	.56***	.55***	–				
Belief in fatalistic determinism FAD (7)	3.53 (0.97)	−.11	.44***	.77***	.53***	.40***	.69***	–			
Belief in unpredictability FAD (8)	3.77 (0.65)	−.09	.58***	.60***	.56***	.57***	.63***	.67***	–		
Mood (9)	3.49 (0.62)	.09	−.13*	−.42***	−.21***	−.04	−.31***	−.44***	−.31***	–	
Religiosity (10)	4.55 (0.99)	−.02	.42***	.59***	.64***	.36***	.47***	.57***	.37***	−.28***	–
Study 5 (*N* = 249)											
Helping (1)	3.58 (0.86)	–									
Belief in free will FWI (2)	5.29 (1.15)	.39***	–								
Belief in determinism FWI (3)	4.94 (1.43)	.61***	.47***	–							
Belief in dualism FWI (4)	5.12 (1.30)	.58***	.57***	.64***	–						
Mood (5)	3.48 (0.64)	−.40***	−.03	–.33***	−.14*						

*Note.* FWI = Free Will Inventory, Nadelhoffer et al. (2014); FAD = FAD plus Scale, Paulhus and Carey (2011).

* *p* < .05. **p < .01. ***p < .001.

In an additional exploratory analysis, we ran a multiple regression with all the FWI subscales as well as mood as predictors and intended helping behavior as the dependent variable (see Table 2 for a full overview). When entering all predictors into the regression equation, belief in dualism remained the only significant predictor of intended helping behavior, β = 0.26, *t* = 4.86, *p <* .001, 95% CI = [.14, .33].

**Table 2.** Regression Table in Study 1.

Model		Unstandardized coefficients		Standardized coefficients	*t*	*p*	95% CI	
	Predictor	*B*	*SE*	β			Lower	Upper
1	(Intercept)	4.41	0.51		8.71	< .001	3.42	5.41
	Free Will FWI	0.05	0.05	0.05	0.88	.379	−0.06	0.15
	Determinism FWI	−0.09	0.05	−0.10	−1.84	.067	−0.18	0.01
	Dualism FWI	0.23	0.05	0.26	4.86	< .001	0.14	0.33
	Mood	0.23	0.13	0.10	1.80	.072	−0.02	0.48

*Note.* CI = confidence interval; FWI = Free Will Inventory.

### Discussion

Study 1 was able to conceptually replicate the results originally found by Baumeister et al. (2009) as belief in free will correlated with intended helping behavior. Going one step further, the results also revealed that belief in dualism better predicted helping than belief in free will and belief in determinism. To replicate this finding, we conducted Study 2. The goal of Study 2 was to test whether we would find similar findings as Baumeister et al. (2009) even when slightly deviating from the original stimulus material, that is, by assessing a new dependent variable.

## Study 2

### Method

Study 2 was preregistered at Aspredicted (https://aspredicted.org/wn2ct.pdf).

#### Participants

Based on the recommendations for stable correlations put forward by Schönbrodt and Perugini (2013), we aimed to recruit at least 250 participants. The final sample consisted of 359 participants (239 female, 105 male, 4 diverse; 3 preferred to self-describe; 8 preferred not to answer). Ages ranged from 16 to 80 (*M* = 28.36, *SD* = 14.40).

#### Procedure

The procedure of Study 2 was similar to that of Study 1. The only difference concerned the measurement of helping behavior.

As in Study 1, participants first filled in the FWI and the PANAS. Cronbach’s alpha was α = .76 for the free will subscale, α = .74 for the dualism subscale, and α = .71 for the determinism subscale. Cronbach’s alpha for the PANAS was α = .83.

Afterward, we assessed intended helping behavior. In Baumeister et al.’s (2009) Study 2, participants learned that the parents of a student named Katie Banks had been killed in a car accident and that Katie Banks was now solely responsible for the care of her siblings. Afterward, participants indicated how many hours (0–9 or more) they were willing to help Katie Banks. We used a slightly different and perhaps more realistic scenario. Specifically, we told participants that the researcher is currently seeking help as he is in need for volunteers to take part in other psychological studies. Afterward, participants indicated how many hours (0–9 or more) they were willing to help the researcher by taking part in psychological studies. We provide the exact wording of each scenario on the OSF (https://osf.io/twxkz/). After participants indicated how many hours they would be willing to help, they could actually register for a participant database on the next page of the survey.

At the end of the study, participants indicated basic demographics (i.e., age and gender) as in Study 1.

### Results

Preregistered correlations between all measures are reported in Table 2. In line with Study 1, belief in dualism correlated significantly with helping behavior, *r* = .12, *p* = .022, 95% CI = [.02, .22]. Neither belief in free will, belief in determinism, nor mood correlated with helping behavior, *r*s < .08, *p*s > .184.

In a final exploratory analysis, we ran a multiple regression with all the FWI subscales as well as mood as predictors and helping behavior as the dependent variable (see Table 3). When entering all predictors into the regression equation, belief in dualism remained the only significant predictor of helping behavior, β = 0.12, *t* = 2.07, *p =* .039, 95% CI = [.01, .45].

**Table 3.** Regression Table in Study 2.

Model		Unstandardized coefficients		Standardized coefficients	*t*	*p*	95% CI	
	Predictor	*B*	*SE*	β			Lower	Upper
1	(Intercept)	0.70	1.19		0.59	.558	−1.64	3.03
	Free Will FWI	0.07	0.11	0.04	0.63	.530	−0.15	0.30
	Determinism FWI	−0.12	0.12	−0.05	−0.99	.323	−0.35	0.12
	Dualism FWI	0.23	0.11	0.12	2.07	.039	0.01	0.45
	Mood	0.32	0.30	0.06	1.09	.278	−0.26	0.91

*Note.* CI = confidence interval; FWI = Free Will Inventory.

## Study 3

Study 2 conceptually replicated the results found in Study 1 as belief in dualism was the best predictor of intended helping behavior. However, in contrast to Baumeister et al. (2009), we were not able to replicate the finding that belief in free will correlates with intended helping behavior. A reason for this failed conceptual replication could be that we used a different vignette to measure helping behavior. Thus, in Study 3, we applied the same vignette as Baumeister et al. (2009) used. Moreover, we accounted for another shortcoming. While in Studies 1 and 2 the order of the assessed variables was fixed, in Study 3, we randomized the order of all measures to counteract possible order effects.

### Method

#### Participants

Based on the recommendations for stable correlations put forward by Schönbrodt and Perugini (2013), we aimed to recruit at least 250 participants via Amazon’s Mechanical Turk. The final sample consisted of 253 participants (95 females, 157 males, and 1 preferred not to answer). Ages ranged from 18 to 68 (*M* = 36.52, *SD* = 10.96).

#### Procedure

The procedure of Study 3 was similar to that of Study 2 with a few exceptions. First, the order of the assessed variables (i.e., helping, mood, and beliefs related to free will) was random. Second, another difference concerned the measurement of intended helping behavior. That is, in Study 3, we used (as Baumeister et al., 2009) the Katie Banks scenario in which participants learned that the parents of a student named Katie Banks had been killed in a car accident and that Katie Banks was now solely responsible for the care of her siblings. Then, participants had to imagine that Katie Banks lives in their neighborhood. Afterward, they indicated how many hours (0–9 or more) they were willing to help Katie Banks. Third, we measured mood with the version of the PANAS validated by Thompson (2007) and computed a mean mood score as in the previous studies. Cronbach’s alpha for the PANAS was α = .69.

Belief in free will, determinism, and dualism were measured with the FWI (Nadelhoffer et al., 2014) as in Studies 1 and 2. Cronbach’s alpha was α = .83 for the free will subscale, α = .87 for the dualism subscale, and α = .91 for the determinism subscale.

At the end, participants indicated basic demographics (i.e., age and gender) as in our previous studies. We provided the exact wording of each variable on the OSF (https://osf.io/twxkz/).

### Results

Correlations between all measures are depicted in Table 1. As in our previous studies, belief in dualism correlated significantly with helping behavior, *r* = .22, *p* < .001, 95% CI = [.10, .34]. Belief in determinism correlated with intended helping behavior too, *r* = .20, *p* = .002, 95% CI = [.08, .31]. Belief in free will and mood did not correlate with intended helping behavior, *r*s < .09, *p*s > .156.

In a final analysis, we ran a multiple regression with all the FWI subscales as well as mood as predictors and helping behavior as the dependent variable (see Table 4). As in our previous studies, when entering all predictors into the regression equation, belief in dualism remained the only significant predictor of helping behavior, β = 0.20, *t* = 2.38, *p =* .018, 95% CI = [.06, .61].

**Table 4.** Regression Table in Study 3.

Model		Unstandardized coefficients		Standardized coefficients	*t*	*p*	95% CI	
	Predictor	*B*	*SE*	β			Lower	Upper
1	(Intercept)	2.16	1.01		2.15	.032	0.18	4.15
	Free Will FWI	−0.19	0.15	−0.10	−1.26	.210	−0.49	0.11
	Determinism FWI	0.22	0.12	0.14	1.74	.082	−0.03	0.46
	Dualism FWI	0.33	0.14	0.20	2.38	.018	0.06	0.61
	Mood	0.16	0.21	0.05	0.78	.438	−0.25	0.57

*Note.* CI = confidence interval; FWI = Free Will Inventory; *SE* = standard error.

## Study 4

Studies 1 to 3 found a stable correlation between helping and belief in dualism, while the relation with belief in free will and determinism was less clear. Since Baumeister et al. (2009) assessed the FAD scale (Paulhus & Carey, 2011) to measure belief in free will, but we used the FWI scale (Nadelhoffer et al., 2014), one may argue that the selection of the measurement instrument (FAD vs. FWI) explains the differences between Baumeister et al.’s findings and ours. Specifically, participants might have been primed with the concept of determinism in the Baumeister et al. study, as the FAD scale does not assess dualism but measures free will and several forms of determinism, with more items on determinism than free will. As the FWI has an equal number of items on free will, determinism, and dualism, participants in our studies might have been put into a somewhat different context. To test whether the difference between the scales might explain the fact that helping is not related to belief in free will in our studies, we conducted Study 4 in which we assessed the FAD first and the FWI at the very end of the study.

In addition, it might be that the relation between belief in dualism and helping is explained by religiosity. In principle, it could be that religious people are more helpful because religion encourages them to help others. Given that the belief in a soul is part of different religions and since belief in dualism is related to the belief in a soul-like construct that survives bodily death (Bering, 2006; Boyer, 2001), it might not be the belief in dualism but religiosity that drives the found effects. In Study 4, we tested whether belief in dualism still predicts helping when we control for religiosity and beliefs related to free will.

### Method

Study 4 was preregistered at Aspredicted (https://aspredicted.org/sa7qi.pdf).

#### Participants

In line with the recommendations for stable correlations put forward by Schönbrodt and Perugini (2013) we aimed for a sample of 250 participants. We recruited participants via Amazon’s Mechanical Turk. 251 participants (94 female, 157 male) took part in the study. Ages ranged from 20 to 69 (*M* = 36.23, *SD* = 11.21).

#### Procedure

After being welcomed to the study and providing informed consent, participants engaged in a Completely Automated Public Turing test to tell Computers and Humans Apart (Captcha). When it was confirmed that participants were human, they filled in the FAD plus scale (Paulhus & Carey, 2011). The FAD plus measures beliefs surrounding free will along four different dimensions on 5-point rating scale (1 = *strongly disagree*; 5 = *strongly agree*). The free will subscale measures belief in free will, but also personal responsibility. The items related to free will measure autonomy but also the declaration that people are responsible for their actions (sample item: “People can overcome obstacles if they truly want to”). The fatalistic determinism subscale captures belief in “destiny” or “fate” (sample item: “Fate already has a plan for each of us”). The unpredictability subscale measures the belief that events in the world are random, unpredictable, and due to luck (sample item: “Life is hard to predict because it is almost totally random”). Finally, people loading high on the scientific determinism subscale believe that events and behaviors are due to biological forces (e.g., “People’s biological makeup influences their talents and personality”) as well as environmental forces (e.g., “Science has shown how your past environment created your current intelligence and personality”). In our sample, Cronbach’s alpha for the different subscales were α = .79 for the free will subscale, α = .80 for the scientific determinism subscale, α = .85 for the fatalistic determinism subscale, and α = .78 for the unpredictability subscale.

After filling in the FAD plus, participants indicated their mood by filling in the PANAS version validated by Thompson (2007; Cronbach’s α =.64) as in Study 3. Subsequently, they indicated their willingness to help Katie Banks in the same way as in Study 3.

Afterward, we measured religiosity with the Religious Worldviews Scale (RWS; Goplen & Plant, 2015) by letting participants indicate their agreement with 19 statements on 7-point rating scales (1 = *strongly disagree*; 4 = *neither agree nor disagree*; 9 = *strongly agree*). To compute an overall score of religiosity, we averaged across all items (Cronbach’s α = .90).

At the end, we assessed beliefs in free will, dualism, and determinism with the FWI (Nadelhoffer et al., 2014). Cronbach’s alpha was α = .64 for the free will subscale, α = .84 for the dualism subscale, and α = .90 for the determinism subscale. Finally, participants indicated their age and gender.

### Results

In line with our preregistration, we first ran correlational analyses (for a full overview, see Table 1). Interestingly, helping did not correlate with any of the assessed predictors based on conventional levels of significance.

Similar to our previous studies, we ran a multiple regression with helping as the dependent variable and all scales (i.e., all the FWI subscales, all FAD plus subscales as well as mood and religiosity) as predictors (for an overview, see Table 5). When entering all predictors into the regression, belief in determinism, β = −0.33, *t* = −2.55, *p =* .012, 95% CI = [−.93, −.12], and belief in dualism, β = 0.21, *t* = 1.97, *p =* .050, 95% CI = [−.00, .81], (both measured with the FWI) remained the only significant predictors of helping behavior.

**Table 5.** Regression Table in Study 4.

Model		Unstandardized coefficients		Standardized coefficients	*t*	*p*	95% CI	
	Predictor	*B*	*SE*	β			Lower	Upper
1	(Intercept)	4.22	1.56		2.70	.007	1.14	7.30
	Free Will FWI	0.35	0.25	0.15	1.39	.165	−0.15	0.85
	Determinism FWI	−0.53	0.21	−0.33	−2.55	.012	−0.93	−0.12
	Dualism FWI	0.41	0.21	0.21	1.97	.050	−0.00	0.81
	Free Will FAD	−0.47	0.35	−0.14	−1.36	.175	−1.16	0.21
	Scientific Determinism FAD	0.51	0.34	0.16	1.49	.136	−0.16	1.19
	Fatalistic Determinism FAD	0.01	0.28	0.00	0.02	.986	−0.55	0.56
	Unpredictability FAD	−0.41	0.36	−0.12	−1.15	.251	−1.12	0.29
	Mood	0.10	0.27	0.03	0.38	.703	−0.42	0.63
	Religiosity	0.02	0.21	0.01	0.10	.919	−0.39	0.43

*Note.* CI = confidence interval; FWI = Free Will Inventory; FAD = FAD plus Scale; *SE* = standard error.

### Discussion

In Study 4, we tested whether assessing the FAD plus at the beginning of the study primes the concept of determinism and, as a consequence, diminishes the correlation between helping and belief in free will. In addition, Study 4 investigated whether belief in dualism is a significant predictor of helping when controlling for religiosity. The results demonstrate that even when assessing the FAD plus first, belief in free will does not predict helping. However, it seems that assessing the FAD plus at the beginning of the study reduces the correlation between helping and belief in dualism. Nevertheless, when entering all assessed scales into a multiple regression, belief in dualism predicted helping even when controlling for religiosity, mood, as well as beliefs related to free will and determinism assessed with the FAD plus and the FWI scale.

## Study 5

Studies 1 to 4 revealed that despite some variability in the predictability of helping, belief in dualism is the most stable predictor. One may argue that a caveat of these studies is that they merely assessed intended helping behavior in hypothetical scenarios. To get a better idea of whether beliefs surrounding free will predict actual helping behavior, we assessed participants’ previous helping behavior in concrete situations in the past.

### Method

#### Participants

As in our previous studies, we aimed to recruit 250 participants via Amazon’s Mechanical Turk. 249 participants (94 female, 155 male) took part in the study. Ages ranged from 20 to 69 (*M* = 36.32, *SD* = 10.55).

#### Procedure

After being welcomed to the study and providing informed consent, participants engaged in a Completely Automated Public Turing test to tell Computers and Humans Apart (Captcha). When it was confirmed that participants were human, they filled in the Free Will Inventory (FWI; Nadelhoffer et al., 2014). Cronbach’s alpha was α = .86 for the free will subscale, α = .86 for the dualism subscale, and α = .90 for the determinism subscale.

Afterward, we assessed mood with the PANAS validated by Thompson (2007) as in our previous studies. Cronbach’s alpha was α = .66.

In Study 5, we administered an adapted version of the Rushton et al. (1981) altruistic personality and the self-report altruism scale to measure participants’ helping behavior in the past. Specifically, participants indicated on 5-point rating scales (1 = *never*; 2 = *once*; 3 = *more than once*; 4 = *often*; 5 = *very often*) how often they had provided help for the following situations in the past: “I have helped push a stranger’s car that was stuck,” “I have given directions to a stranger,” “I have made change for a stranger,” “I have helped carry a stranger’s belongings (e.g., boxes, suitcase etc.),” “I have given a stranger a lift in my car,” “To help a neighbor whom I didn’t know too well, I have let him or her borrow an item of some value to me (e.g., a dish, tools, etc.),” “I have offered to help a handicapped or elderly stranger cross a street,” and “I have helped an acquaintance to move households.” To compute a helping score, we averaged across all items (Cronbach’s α = .90). Finally, participants indicated their age and gender.

### Results

As in our previous studies, we first correlated helping with all assessed measures. As can be seen in Table 1, belief in free will (*r* = .39, *p* < .001, 95% CI = [.28, .49]), belief in determinism (*r* = .61, *p* < .001, 95% CI = [.53, .68]), and belief in dualism (*r* = .58, *p* < .001, 95% CI = [. 49, .65]) significantly correlated with previous helping behavior.

Entering all predictors (i.e., belief in free will, belief in determinism, belief in dualism, and mood) into a multiple regression (see Table 6) revealed that belief in dualism (β = 0.32, *t* = 4.91, *p <* .001, 95% CI = [.13, .30]), belief in determinism (β = 0.29, *t* = 4.54, *p <* .001, 95% CI = [.10, .25]), and mood (β = −0.26, *t* = −5.30, *p <* .001, 95% CI = [−.48, −.22]) remained significant predictors of helping, while belief in free will did not (β = 0.07, *t* = 1.21, *p =* .226).

**Table 6.** Regression Table in Study 5.

Model		Unstandardized coefficients		Standardized coefficients	*t*	*p*	95% CI	
	Predictor	*B*	*SE*	β			Lower	Upper
1	(Intercept)	2.57	0.32		7.93	< .001	1.93	3.21
	Free Will FWI	0.05	0.04	0.07	1.21	.226	−0.03	0.14
	Determinism FWI	0.18	0.04	0.29	4.54	< .001	0.10	0.25
	Dualism FWI	0.21	0.04	0.32	4.91	< .001	0.13	0.30
	Mood	−0.35	0.07	−0.26	−5.30	< .001	−0.48	−0.22

*Note.* CI = confidence interval; FWI = Free Will Inventory; *SE* = standard error.

## Mini Meta-Analysis Across All Five Studies

The validity of the different beliefs in predicting helping depended to some degree on the study carried out. To get further insights into the relation between beliefs associated with free will and helping, we conducted a random effects meta-analysis across all five studies consulting the MAVIS Meta-Analysis via Shiny software (Version 1.1.3; Hamilton et al., 2016). For each belief, we ran a separate mini meta-analysis. The meta-analysis revealed that belief in determinism is not correlated with helping (*r* = .14, *Z* = 0.98, *p* = .327). Mood was not correlated with helping either (*r* = −.03, *Z* = −0.28, *p* = .783). More important for the purpose of the present article, a further meta-analysis revealed that belief in free will is significantly (although weakly) related to helping behavior (*r* = .14 *Z* = 2.16, *p* = .031, 95% CI = [.01; .26]). The meta-analytical effect for belief in dualism was stronger in size and significance too (*r* = .26, *Z* = 2.59, *p* = .01, 95% CI = [.06; .43]). In a final analysis, we tested whether the correlation between belief in dualism and helping was stronger than the correlation between belief in free will and helping. In a first step, we calculated for each study the effect size for the comparison between the correlation of belief in dualism and helping versus the correlation of belief in free will and helping in accordance with the calculation proposed by Eid et al. (2011). In a second step, we ran the meta-analysis by entering the resulting effect sizes into the MAVIS Meta-Analysis via Shiny software (Version 1.1.3; Hamilton et al., 2016). A random effects meta-analysis revealed that the correlation between belief in dualism and helping was stronger than the correlation between belief in free will and helping (*Z* = 3.07, *p* = .002, 95% CI = [.04; .18]).

## General Discussion

Within the last decades, psychologists started to investigate the consequences of laypeople’s beliefs surrounding free will (for a review, see Ewusi-Boisvert & Racine, 2018). One of the most influential articles in this literature is Baumeister and colleagues’ (2009) paper showing that belief in free will relates to individuals’ willingness to help others. However, since recent research started to cast doubt on the general link between free will beliefs and social behavior (e.g., Genschow et al., 2022), the question arises as to whether Baumeister et al.’s findings would withstand a high-powered conceptual replication. To answer this question, we conceptually replicated and extended Baumeister et al.’s Studies 1 and 2. As belief in free will and belief in dualism are closely linked to each other (Wisniewski et al., 2019), we investigated whether belief in free will or belief in dualism better predicts intended helping behavior. Meta-analytical evidence across all studies indicates that belief in determinism is not correlated with helping. More importantly, the meta-analysis supports Baumeister et al.’s findings as belief in free will correlated with helping behavior. Interestingly, belief in dualism correlated more strongly with helping behavior. Moreover, in each study, we found that even when statistically controlling for belief in free will, belief in determinism, and mood, belief in dualism remained a significant predictor of intended helping behavior. These findings have important theoretical implications despite some limitations.

### Implications and Future Directions

Previous research found that exposing participants to anti-free will messages influences social behavior (e.g., Baumeister et al., 2009; Vohs & Schooler, 2008). However, recent non-successful replications questioned these original findings (Monroe et al., 2017; Nadelhoffer et al., 2020; Open Science Collaboration, 2015; Schooler et al., 2014; Zwaan, 2014). Based on a recent meta-analysis (Genschow et al., 2022), a potential explanation for the mixed results is that current experimental manipulations are not able to influence social behavior because these manipulations produce rather weak and unspecific effects. Thus, it might well be that although experimental studies are not able to support the idea that free will beliefs are related to social behavior, this relation does nevertheless exist on an interindividual level. To test this explanation, we conceptually replicated Baumeister et al.’s (2009) Studies 1 and 2. Overall, the results replicate the results obtained by Baumeister et al. as individuals’ belief in free will correlated with their willingness to help others. This finding is in line with recent studies indicating that on a correlational level belief in free will is related to different meaningful behaviors such as punishment for unethical behavior (Clark et al., 2014; Genschow et al., 2017; Martin et al., 2017), reward for ethical behavior (Genschow et al., 2017), the tendency to blame victims for their wrongdoings (Genschow & Vehlow, 2021), or the perception of intentionality in others’ (Genschow et al., 2019) and one’s own (Genschow & Lange, 2022) behaviors. At the same time, our findings suggest that it is mainly belief in dualism (instead of free will) that predicts helping. Future research should, thus, test whether other instances involving belief in free will and correlations with social behavior are in fact driven by belief in dualism.

On a conceptual level, dualism is often considered one of the cornerstones of complex beliefs such as the belief in gods and religious belief as a whole (Bloom, 2007). Indeed, people believing in dualism typically believe in the existence of a soul-like construct that survives bodily death (Bering, 2006; Boyer, 2001). This belief together with the belief that moral traits are manifested in one’s soul (Strohminger & Nichols, 2014) could explain why people believing in dualism aim at behaving in a moral and pro-social fashion. Thus, it might well be that other pro- and anti-social behaviors that have been previously linked to free will beliefs are driven by belief in dualism also.

The finding that belief in dualism is a key predictor of helping offers a new perspective on the question of why people help. Previous theories on the relation between beliefs surrounding free will and helping assume that moral responsibility is the key driver of the relation between free will beliefs and pro-social behavior (Shariff et al., 2014). Our finding that it is especially the belief in dualism, and thereby the belief in souls, that relates to helping extends previous theoretical considerations by suggesting that people may ultimately have a self-interested, if not selfish, motive to help. That is, people may decide to help others because they think it would be good for their immortal souls and not necessarily because they feel a moral responsibility. Future research should test this interpretation more directly.

### Limitations

Despite these implications, we need to acknowledge some limitations. First, Studies 2 and 5 assessed intended helping behavior in a somewhat different manner than Baumeister et al. (2009) did. Thus, one could argue that the paradigms we used are not ideal for conceptually replicating Baumeister et al. However, in Study 3 we used the exact same paradigm as Baumeister et al. and found similar results. Thus, we regard it as rather unlikely that the paradigms alone account for our effects.

Second, as all of our studies applied a correlational design, we cannot draw any causal inferences. Indeed, it might well be that belief in dualism does not influence helping but that helping influences participants’ belief in dualism. Future research is advised to develop valid and reliable experimental methods—which are currently missing in the literature—to manipulate participants’ belief in dualism and to test whether manipulated beliefs in dualism change participants’ helping behavior.

Third, an important shortcoming is the free will belief scales we used. A particular characteristic of all the free will belief scales available in the literature is that they do not include any reversed item. Thus, in principle, it might be that the found correlations merely represent the tendency to agree with a variety of different statements. At the same time, however, such an explanation of our results does not explain why in all of our studies belief in dualism remained a significant predictor even when controlling for other beliefs. Also, it does not explain why we also found some negative correlations. Nevertheless, future research is advised to develop new scales that include reverse-formulated items.

Finally, a somewhat surprising observation is that the correlations between the different subscales were stronger in Studies 3 to 5 as compared with Studies 1 and 2. There might be different reasons for this. On the one hand, Studies 1 and 2 were conducted in Germany, while Studies 3 and 5 were MTurk samples from the United States. It might be that cultural differences account for the fact that in some studies the scales correlated more strongly than in other studies. On the contrary, it could be that MTurkers are more experienced in filling in questionnaires, which may account for the strong intercorrelations. In any case, these explanations are based on speculations that should be tested in future research.

## Summary

The present study replicated Baumeister et al.’s (2009) finding by demonstrating that belief in free will correlates with helping. At the same time, our studies extend previous research by indicating that it is not the belief in free will per se, but rather the belief in dualism that best predicts helping.
